# Improved Efficiency and Reliability of NGS Amplicon Sequencing Data Analysis for Genetic Diagnostic Procedures Using AGSA Software

**DOI:** 10.1155/2016/5623089

**Published:** 2016-08-30

**Authors:** Axel Poulet, Maud Privat, Flora Ponelle, Sandrine Viala, Stephanie Decousus, Axel Perin, Laurence Lafarge, Marie Ollier, Nagi S. El Saghir, Nancy Uhrhammer, Yves-Jean Bignon, Yannick Bidet

**Affiliations:** ^1^Département d'Oncogénétique, Centre Jean Perrin, 63011 Clermont-Ferrand, France; ^2^EA 4677 ERTICa, Université Clermont Auvergne, 63000 Clermont-Ferrand, France; ^3^Naef K. Basile Cancer Institute (NKBCI), Breast Center of Excellence, American University of Beirut Medical Center, Beirut 1107 2020, Lebanon

## Abstract

Screening for* BRCA *mutations in women with familial risk of breast or ovarian cancer is an ideal situation for high-throughput sequencing, providing large amounts of low cost data. However, 454, Roche, and Ion Torrent, Thermo Fisher, technologies produce homopolymer-associated indel errors, complicating their use in routine diagnostics. We developed software, named AGSA, which helps to detect false positive mutations in homopolymeric sequences. Seventy-two familial breast cancer cases were analysed in parallel by amplicon 454 pyrosequencing and Sanger dideoxy sequencing for genetic variations of the* BRCA *genes. All 565 variants detected by dideoxy sequencing were also detected by pyrosequencing. Furthermore, pyrosequencing detected 42 variants that were missed with Sanger technique. Six amplicons contained homopolymer tracts in the coding sequence that were systematically misread by the software supplied by Roche. Read data plotted as histograms by AGSA software aided the analysis considerably and allowed validation of the majority of homopolymers. As an optimisation, additional 250 patients were analysed using microfluidic amplification of regions of interest (Access Array Fluidigm) of the BRCA genes, followed by 454 sequencing and AGSA analysis. AGSA complements a complete line of high-throughput diagnostic sequence analysis, reducing time and costs while increasing reliability, notably for homopolymer tracts.

## 1. Background

Mutations in the* BRCA1* and* BRCA2* genes are associated with high risk of breast and ovarian cancer [1, 2]. Mutation screening in families with multiple breast and/or ovarian cancer cases (hereditary breast and ovarian cancer (HBOC)) or exceptionally young cases has revealed a high frequency of germline mutations in these genes [3, 4]. To detect these mutations, genomic DNA sequencing remains the gold standard, as pathogenic variants can occur throughout the gene and the entire gene coding sequence must therefore be screened. Historically performed by PCR amplification and Sanger dideoxy sequencing,* BRCA* resequencing is time-consuming and expensive. The major technological advances of new generation sequencing provide high-throughput strategies to reduce the time and expense of direct sequence analysis [5–7]. Furthermore, various methods of multiplex or microfluidic PCR reduce the time necessary to prepare samples before sequencing.

Among massively parallel sequencers, the GS-FLX and GS-Junior (Roche) and the PGM and Proton (Life Technologies) have the advantage of generating read lengths of up to 500 bases. Amplicons resembling or identical to those currently used for dideoxy sequencing can thus be analysed. On the other hand, these sequencers have the inconvenience of generating numerous reading errors in homopolymeric sequences, which complicates the analysis. Many software approaches have been developed to correct NGS calling errors [8]. Some produce good results, but false indel detection in homopolymers remains challenging. Moreover, these software solutions often require significant computational power and bioinformatics skills that are difficult to maintain in a routine medical diagnostic laboratory.

This study addresses the suitability of pyrosequencing technology associated with in-house developed software for molecular diagnosis, in terms of performance, robustness, and reliability. In preparation for high-throughput analysis of HBOC families, our results on cases blindly analysed with both techniques validate sequencing on the GS-FLX for mutation detection in the* BRCA1* and* BRCA2* genes.

## 2. Methods

### 2.1. Family Selection

72 HBOC family samples used for technical comparisons were identified in the Oncogenetics consultation of the Centre Jean Perrin. 250 HBOC samples used for microfluidic amplification were identified in the Oncogenetics consultation of the Naef K. Basile Cancer Institute (NKBCI) at the American University of Beirut Medical Center (AUBMC, Beirut, Lebanon). DNA was isolated from peripheral blood by standard techniques, using NucleoSpin® Blood XL kit (Macherey-Nagel) at the Centre Jean Perrin and QIAmp DNA isolation kit (Qiagen) at the AUBMC.

### 2.2. Ethical Approval and Consent

All patients gave informed consent for analysis of breast cancer predisposition genes. Lebanese HBOC patients were recruited in a study of* BRCA1* and* BRCA2* mutations in Lebanon approved by IRB at AUBMC, granted by Ethnic Research Initiative (ERI) and sponsored by GlaxoSmithKline (GSK).

### 2.3. Amplicon Design

The amplicons traditionally used for Sanger dideoxy sequencing by our laboratory for the analysis of the* BRCA* genes ranged from 195 to 592 base pairs and included the entire coding sequence plus all intron-exon junctions. The* BRCA1* coding sequence (7,224 bp) was covered in 31 amplicons (10,823 nucleotides of sequence) and* BRCA2* (11,386 bp) in 44 amplicons (16,513 nucleotides of sequence). Large exons were covered by overlapping amplicons. For dideoxy sequencing, all forward primers carried an M13 forward extension and all reverse primers an M13 reverse extension.

For GS-FLX pyrosequencing, amplicons exceeding 500 bp were redesigned, resulting in 40 amplicons for* BRCA1* (10,006 nucleotides of sequence) and 44 for* BRCA2* (15,396 nucleotides of sequence), from 142 to 501 bp in length. The 454 “A” and “B” sequencing primer extensions were included at the 5′ ends of all forward and reverse PCR primers, respectively. Primers were also designed to homogenize amplification conditions.

For more uniform coverage, some poorly represented sequences were amplified in duplicate in the Access Array.

### 2.4. Sanger Dideoxy Sequencing

Amplicons were sequenced by standard techniques, using BigDye v3 (Applied Biosystems) reagents, and were resolved on a 3130XL capillary sequencer (Applied Biosystems). Sequences were aligned with reference sequences NM_007294.2 (*BRCA1*) and NM_000059.3 (*BRCA2*) using Seqman software (DNAStar). All variants were noted; deleterious mutations were confirmed by independent analysis of second samples.

### 2.5. Pyrosequencing

For standard amplification, 72 amplicon libraries were prepared from genomic DNA by amplification in 96-well plates, one plate per patient. After verification on agarose gels and AmpureXT purification according to Beckman Coulter's recommendations, amplicons were quantified with PicoGreen (Invitrogen) on an Infinite 200 plate reader (Tecan), according to Invitrogen's guidelines. Isomolar amplicon pools were prepared for each sample, and these libraries were diluted to 2 × 10^5^ molecules/*µ*L.

For microfluidic amplification, we used the Access Array System (Fluidigm) according to the manufacturer's instructions. This technology allows amplification of 48 amplicons for 48 patients at once. During the same PCR, Roche MID oligonucleotide adapters were added to allow multiplexing and identification of patients.

Emulsion-PCR, bead recovery and enrichment, and pyrosequencing were performed as recommended (emPCR LibA and sequencing kit, Roche).

### 2.6. Data Processing

Pyrosequencing data processing was performed using the software provided by Roche: raw images were automatically converted into sequences by gs Run Processor v2.3 and the sequences were aligned to the* BRCA* references by Amplicon Variant Analyzer (AVA v2.3). AVA uses a package called “newbler” developed specifically for 454 sequencing that does both alignment and variant calling [9]. It is then able to display variants graphically, with a histogram indicating both the number of reads at each position and the percentage of variant reads. Variations are also accessible with a color-coded multiple alignment which highlights regions and bases differing from the reference sequence. Read frequencies of variants were reported in a summary table for each sample.

### 2.7. Data Analysis

We developed a Java pipeline called AGSA which takes as input a calculated AVA project and the Genbank file of the gene(s) of interest. AGSA was developed to detect and annotate variants and generate easy-to-read results in a color-coded Excel result file.

Results were validated according to several adjustable parameters, such as read depth, the percentage of reads presenting a variant, and the presence of variants on both strands.

The minimum read depth was set to 40 for each nucleotide to validate an amplicon, and the minimum percentage of mutated reads to 20% by default (however, a variant can be detected in a nonvalidated amplicon). Furthermore, it was possible to specify the region of interest for each exon (default value −20 to +6 to include splicing sites). For a relevant comparison, the same settings were used in the SeqNext Software. HGVS nomenclature was obtained for each variant thanks to a request to the Alamut servers (Interactive Biosoftware, Rouen, France). After this annotation, a homopolymer analysis step was added to help users decide if a detected indel variant was an artefact due to the technology or a real variant.

### 2.8. Homopolymer Analysis

The homopolymer analysis consists of the construction of a histogram showing two representations of the same dataset, for each putative variant located in a homopolymer. The distribution of the measured light intensity at the position considered (flowgram values) was represented in blue with a step of 1 to mimic AVA's interpretation of the sequence and in red with a step of 0.1 (Figure 2). Datasets were extracted from the sff files located in the AVA project.

### 2.9. Presentation of “AGSA” Software

**Figure 1:**
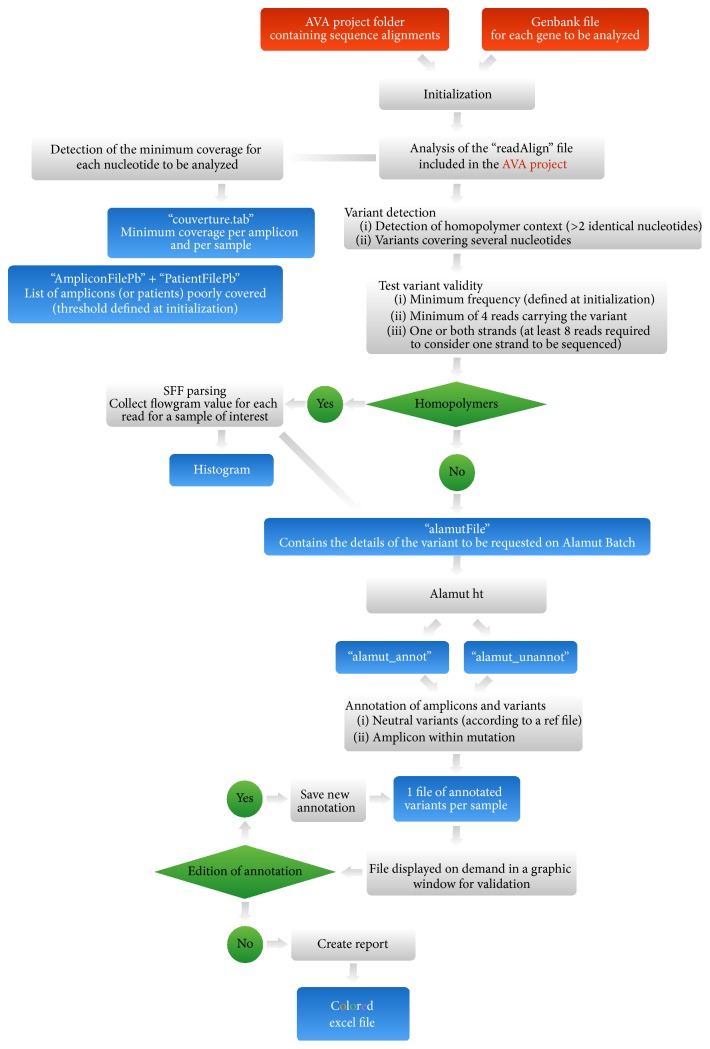
Organization of the AGSA software. The diagram represents the operational flow of the AGSA software. The red boxes represent input files required to operate the software. The blue boxes represent output files generated by AGSA. The green boxes are steps where the software performs a test.

**Figure 2:**
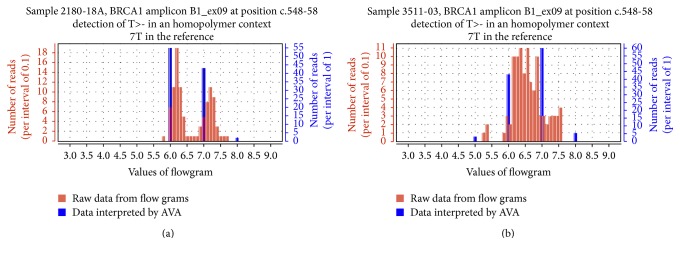
Graphic representations of homopolymer flowgrams. Individual flowgrams of indel variants were generated by AGSA. The *X*-axis represents the signal intensity computed during pyrosequencing. Red bars represent the number of reads for each intensity interval of 0.1; blue bars represent the percentage of variation as shown by AVA (intensity interval of 1). Distribution of standard flowgrams discriminates real indel (a) from artefacts (b).

AGSA was developed in order to facilitate 454 sequencing analysis of one or more gene(s) of interest. AGSA is a graphic interface software with few parameters to adjust: boundaries of the region of interest for each exon, threshold value for the minimum depth to validate an amplicon, and threshold frequency to accept a variant (screenshot in Supplementary Figure  1A, in Supplementary Material available online at http://dx.doi.org/10.1155/2016/5623089). To keep it simple, AGSA uses Amplicon Variant Analyser (AVA) output files as raw data and the Genbank file(s) of the studied gene(s) (Figure 1). During processing, several output files are created to control the sequencing quality by the coverage of each amplicon and each sample. Read depth is determined for each base of the region of interest, but only the minimum is used to consider an amplicon correctly sequenced or not. Detection of variants is calculated independently of read depth, to make sure that variants are not missed, even in poorly sequenced amplicons that should be repeated. To be validated and kept in the final report, a variant must be present on both strands (unless there were reads for only one strand) and in at least 4 reads (to eliminate random sequencing errors). The variant must also exceed the minimum percentage of reads defined in the parameters. These settings permit mutation calling in insufficiently covered amplicons, without generating many false positives. Then the program tests the nucleotides just before and after the position of the variant, to check if it is in a homopolymer context. When the variant is detected within a homopolymer, AGSA searches the sequence flowgram file for the values of all reads of the sample at the variant position. A histogram is created and saved as a  .jpeg file. For a heterozygous insertion or deletion, the distribution of read values is split into two populations, showing that some reads have *n* identical bases and others *n* + 1 (insertion) or *n* − 1 (deletion) identical bases (Figure 2(a)). On the contrary, in case of artefacts, a single population is observed between *n* and *n* + 1 (or *n* − 1), resulting from poor quantification of the strong homopolymer signal (Figure 2(b)). In case of homozygous variation, a single population is observed too but it is centred on *n* + 1 (or *n* − 1), showing that all reads have the same number of bases in the homopolymer and that this number is different from the wild type. The graphs aid the interpretation of the data as one or two different alleles; the mono- or bimodal distribution of flowgram values may also be evaluated statistically (though we have not yet developed this function). AGSA extracts and graphs the relevant signal information from the raw data for all detected variants in a homopolymer context, for each sample. Variant annotation is then performed using Alamut Batch software v1.1.6 (Interactive Biosoftware, Rouen, France). Annotation includes the nomenclature recommended by the Human Genome Variation Society (HGVS), as well as predictions about the mutation impact determined by different algorithms. Finally, AGSA automatically annotates variants identified as neutral in a list supplied by the users (here the BIC and UMD-*BRCA* databases were used) and also reports the amplicons without variants. Any variants that have not been automatically classified must be interpreted manually by the user. To assist this step we developed a graphic interface with all information needed to annotate the variant (position, homopolymer context, graphic of homopolymer, variant frequency, coverage depth, and so forth, Supplementary Figure  1B). When all variants are sorted, the user can generate a report to summarize the annotation of the gene(s) of interest for all samples.

This software was created in interaction with end-users in order to simplify the software utilization and graphic interface. It is thus an easy-to-use tool, with very few parameters to manage. AGSA software was initially developed to analyse* BRCA1* and* BRCA2* for diagnosis purposes, but any combination of genes can be analysed.

## 3. Results and Discussion

### 3.1. Comparison of* BRCA* Analysis Using Sanger Sequencing and Roche Pyrosequencing with AGSA Analysis

One run of 8 samples and four runs of 16 samples for a total of 72 different patients were performed using GS-FLX chemistry. These 72 samples were analysed in parallel by Sanger dideoxy sequencing. All 72 samples were coded for blind analysis. Dideoxy sequencing and pyrosequencing were performed by different technicians. The Titanium technology was developed to produce 400 bp long reads. Although it was able to produce longer reads, a random sampling of 10% of the samples showed that the quality dropped quickly after 400 nucleotides (Supplementary Figure  2). 77% of nucleotides of the 400 bp long reads presented a quality score above *Q*20, which is the threshold used for Sanger sequencing, and 53% were above *Q*30. The longest fragments were covered on both ends by high quality base calls, with a large overlap in the middle.

Our own observations, as well as discussions with other groups and expert committees [7, 10], suggested limits for the analysis of pyrosequencing data. Amplicons were validated when no nucleotide in the region of interest was read less than 40 times, and heterozygous variants were validated when present in at least 20% of reads and represented on both strands. This latter threshold is lower than the smallest value found for the known mutations of the composite sample described below (see Section 3.3).

**Figure 3:**
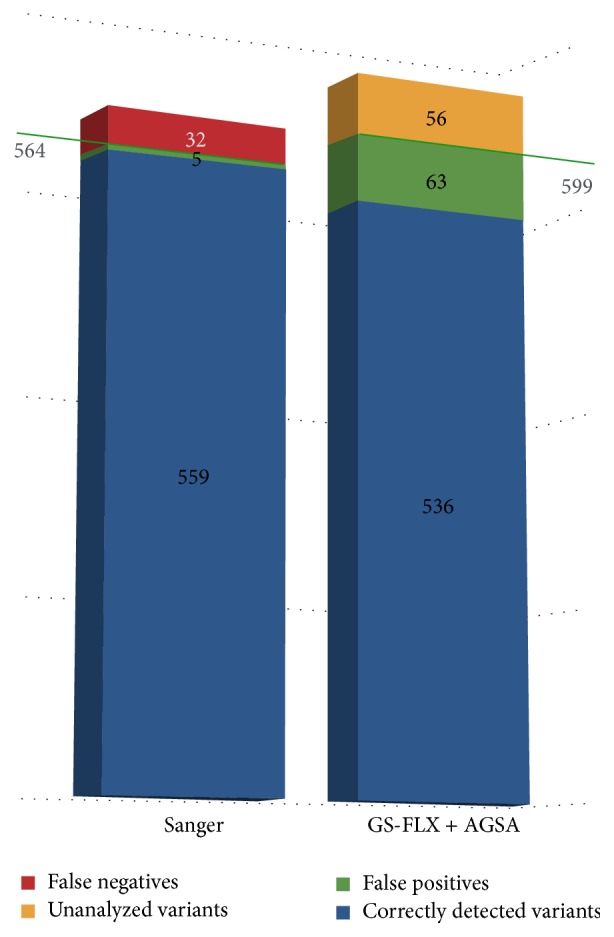
AGSA detected efficiently all the variants reported in Sanger analysis. Efficiencies of Sanger sequencing versus Roche pyrosequencing analysed with AGSA. The blue bars represent confirmed variants. The green bars represent false positive variants (technical artefacts). For pyrosequencing, false positives are defined as variants not confirmed by Sanger sequencing. For Sanger sequencing, false positives were not found by pyrosequencing and were not confirmed by a second Sanger run of the same sample. The red bars represent false negative variants. No false negative was found by pyrosequencing. False negatives for Sanger analysis were detected by pyrosequencing and they were actually found on a second Sanger run of the same sample. The yellow bar represents variants that were not called by AGSA because of poor coverage (inducing a number of variant reads < 4).

Because dideoxy sequencing remains the gold standard for diagnostic purposes, variants were classified according to their Sanger status: only variants detected by the method of reference were considered real. 564 variants were reported in the 72 patients tested by dideoxy sequencing (Figure 3). These variants consisted of substitutions and a wide range of insertions/deletions (1 to 29 nucleotides). They included frequent polymorphisms, deleterious mutations, and variants of unknown significance. Pyrosequencing followed by analysis with AGSA software reported 599 variants. As a comparison, analysis only with AVA generated a list of 3800 putative variants (data not shown). Despite the overall higher number of variants called after pyrosequencing, 61 variants detected by the Sanger technique were missing in the pyrosequencing reports. 56 of these were in amplicons not validated due to insufficient read depth and automatically targeted to be resequenced by standard techniques. The remaining 5 variants were Sanger false positives since a second Sanger analysis of these samples revealed a wild-type sequence. These false positives were due to information transfer errors or to poor quality Sanger sequences; none were potentially deleterious mutations.

Conversely, pyrosequencing revealed 95 variants reported by AGSA but not by Sanger analysis. When the Sanger traces were reviewed, 32 variants were actually present and can thus be considered false negatives of Sanger analysis. Most were information transfer errors; some were due to sequences more difficult to interpret. Excepting one unclassified variant in* BRCA2*, all were frequent polymorphisms. In total, pyrosequencing allowed correcting 37 errors in Sanger reports. We insist on the fact that these false positives or negatives were not generated by the Sanger technique itself. They were due to inevitable small rate of human errors when manually analysing large volumes of data in routine diagnostic procedures. The other 63 variants were falsely reported after analysis of the pyrosequencing data. Because Sanger sequencing is the technique of reference, these were considered artefactual variants due to poor quality reads in some amplicons (mainly in* BRCA2*), although, unlikely, some of them could however be real variants not detected by Sanger sequencing.

Nevertheless, most of the false positive variants represent technical limits of the 454 NGS sequencing in homopolymers. These were specifically treated by AGSA software.

### 3.2. AGSA Software Eliminates Most False Positives without Generating False Negatives

One bias of 454 pyrosequencing is the misreading of homopolymeric sequences that generates many false positive variants. To respond to this bias, AGSA software aims to determine if a reported variant in a homopolymer is a reading mistake or a true variant and presents this information to the user as explained above. For example, for a heterozygous deletion in a series of 7 A's, the distribution of raw data falls into two populations, showing that some reads have 7 identical bases and others 6 identical bases (Figure 2(a)). When this same sequence is misread, only one population is observed between 6 and 7 nucleotides resulting from poor quantitation of the strong homopolymer signal (Figure 2(b)). At this time, the classification of the graphs as representative of an artefact (monomodal distribution) or a real variant (bimodal distribution) is determined by the user.

**Figure 4:**
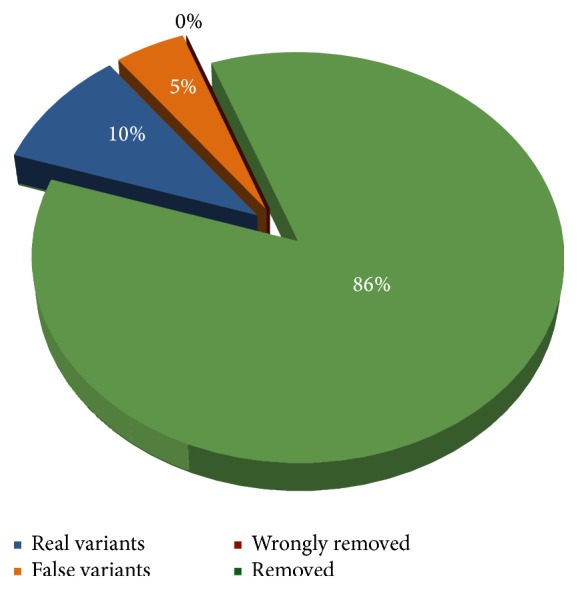
Performance of AGSA software for evaluation of homopolymers. 299 indel variants were found by AGSA in homopolymer sequences. After analysis of individual flowgrams, 246 (86%) were classified as false positive variants and 43 (14%) as true variants. Sanger sequencing confirmed that the 246 AGSA-classified false positives were actually wild-type sequences. Among the 43 potentially real variants, 29 (10%) were confirmed with Sanger analysis and 14 (5%) were actually wild-type Sanger sequences.

To evaluate the accuracy of the result delivered by AGSA, we blindly assessed 299 putative variants in six different homopolymeric sequences (of which one is a common polymorphism with frequent heterozygotes and both classes of homozygotes), detected in our 72 patients. The histograms were interpreted independently by two persons. Although subjective, the interpretation of the histograms generated by AGSA was robust, as more than 90% of the cases were assessed concordantly. Most of the discordances related to one reader being not sure of the interpretation (Supplementary Figure  3). Discordances were not critical, however, as all doubtful sequences are flagged for further confirmation. 256 samples were defined as homozygous wild type and 43 as heterozygous (Figure 4). Dideoxy sequencing confirmed all wild-type classified homopolymers as homozygous. Among the 43 heterozygous calls at the polymorphic homopolymer, 29 were heterozygous and 14 were homozygous.

With help of the homopolymer histograms, 86% of the indel variants in homopolymer contexts were eliminated, while no false negatives were created (Figure 4). Of the remaining variants, 10% were real variants and 5% were false positives.

### 3.3. Comparison of AGSA to SeqNext Software

**Figure 5:**
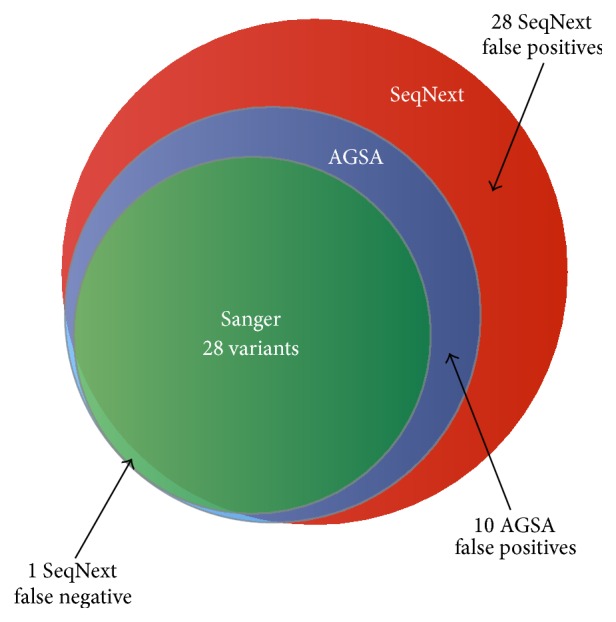
A composite sample including 28 variants validated in Sanger was analysed both with AGSA software and with SeqNext, using the same threshold of 20%. AGSA detected all 28 variants and 10 false positive variants whereas SeqNext missed 1 real variant and reported 28 false positives.

One of the samples analysed in the test phase was a composite sample including 20 deleterious mutations and 8 polymorphisms. This sample was generated by pooling appropriate PCR products from different patients, with known* BRCA* variants ranging from single nucleotide substitutions to a 29-base-pair deletion, including transitions, transversions, single nucleotide insertions and deletions, and a deletion of one nucleotide in a homopolymer of eight. All expected variants were detected by AGSA (Figure 5 and Table 1), as well as 10 false positive variants that were not present in the Sanger analyses. Six of these were in amplicons with less than 40 reads, which could explain loss of specificity. However, seeking variants in amplicons with low coverage allowed detection of two real variants in* BRCA1* (c.19_47del and c.212+1G>A).

To compare the performance of AGSA to commercial software for NGS data analysis, we tested the SeqNext module of Sequence Pilot from JSI Medical Systems (version 4.1.2). To stay close to AGSA parameters and after discussions with the developer, we set parameters to read depth ≥ 40, 20% variant reads, and regions of interest from −20 to +6 per exon. With SeqNext, one false negative and 28 false positive variants were detected (Figure 5 and Table 1). Most of these false positives were in poorly sequenced amplicons. AGSA appears to deal better with these regions. The missed variant was an insertion of 39 nucleotides. According to the developer, it was missed because the sequence downstream of the insertion is too short to allow realignment. In this specific case, mutated reads are ignored. One nomenclature mistake was also detected with SeqNext (c.3839_3844delinsAGGCG instead of c.3839_3843delinsAGGC).

To validate the results obtained on this composite sample, SeqNext analysis was performed on a sampling of 39* BRCA1* and 33* BRCA2* analyses from the 72 test patients (Supplementary Figure  4). SeqNext detected all the variants found by Sanger sequencing. Nevertheless, SeqNext gave about twice as many false positives as AGSA (177 for SeqNext versus 85 for AGSA).

AGSA software thus seems to be more efficient for Roche pyrosequencing analysis as it generates fewer false negative and false positive variants.

### 3.4. Protocol Optimization

To challenge the robustness of our method of* BRCA* pyrosequencing, we performed a larger series of 250 patients. To optimize the cost and duration of the analysis, we used microfluidic amplification on the Access Array Fluidigm system, generating 48 amplicons for 48 patients simultaneously. The analysis of* BRCA1* and* BRCA2* sequences of 250 Lebanese patients was performed using AGSA; variants were confirmed by dideoxy sequencing.

**Figure 6:**
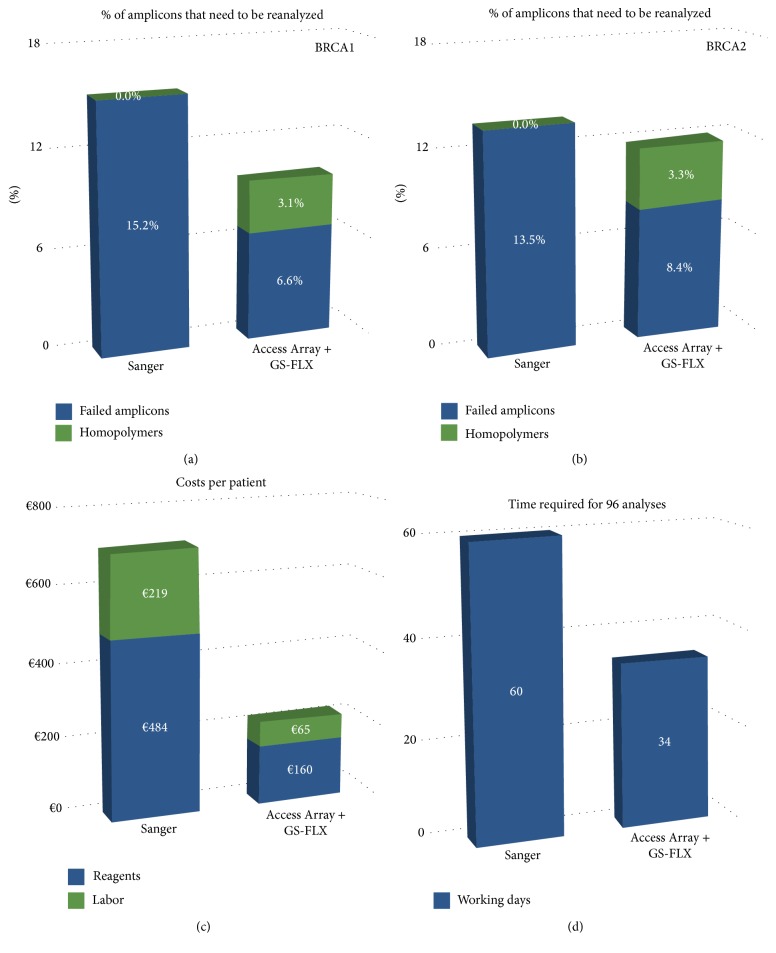
250 patients were studied for* BRCA* mutations by Access Array Fluidigm combined with 454 pyrosequencing and AGSA analysis. This sequencing methodology was compared to Sanger analysis in terms of percentage of amplicons to be reanalysed for* BRCA1* (a) and* BRCA2* (b), cost per patient (c), and time required to analyse 96 patients (d).

We first examined the coverage of each amplicon across the six runs performed. Around 10% of all amplicons had to be repeated by dideoxy sequencing. This included 7.5% that did not reach the 40-read threshold and 3.2% of homopolymers that were not eliminated by AGSA (Figures 6(a) and 6(b)). The percentage of homopolymers flagged for confirmation decreased from the first to last runs (3.2% versus 9% (22 + 5 for 299 variants), Supplementary Figure  3), suggesting that confidence in reading the histograms generated by AGSA increased with experience. With Sanger analysis, the first run of sequencing for each patient resulted in a low percentage of failed amplification or sequencing. In our laboratory this percentage was evaluated on our most recent 500 patients analysed using the Sanger technique, with a mean of 15% in* BRCA1* and 13% in* BRCA2*. Thus Sanger sequencing and Roche pyrosequencing generate similar rates of technical failure, with no correlation between the two techniques for samples either partially or fully analysed by both techniques.

The cost and duration of analyses were both improved. Reagent costs were reduced by 67% and technician time was reduced by 71% (Figure 6(c)). Finally, the analysis of 96 patients was estimated at about 34 working days for GS-FLX sequencing versus 60 for Sanger sequencing (Figure 6(d)). This time saving is essential since the delay for reporting results can be very long for* BRCA* sequencing. With the development of specific cancer therapies targeted to* BRCA* mutation carriers, there is a crucial interest in lowering these turnaround times.

## 4. Conclusions

Although NGS methods are starting to be used routinely in many molecular genetic laboratories [7, 11–13], Sanger dideoxy sequencing remains the gold standard technique. This study aims to validate our NGS method for constitutional genetics diagnosis. This method combines 454 sequencing and analysis with AGSA in-house developed software. Comparing NGS to Sanger sequencing for 72 samples, we showed that all variants found in standard Sanger method were also found by NGS when the conditions of analysis were set to a minimum of 40 reads and 20% of reads carrying a variant. This method is thus at least as sensitive as Sanger sequencing. Moreover we showed that this method allows automation of the sequence reading and thus decreased human potential error rate. This is particularly interesting in a goal of increasing the quality of routine diagnostic procedures.

Some false positive variants in homopolymers were found in 454 pyrosequencing compared to Sanger analyses. This is a recurrent problem for Ion Torrent (Life Technology) and 454 (Roche) sequencing. Several authors developed and tested different analysis workflows in order to correct false indel detections [8, 14–17]. Coral is so far the state-of-the-art error corrector [17]. It is very efficient for substitutions, but it does not account for homopolymer context when interpreting indels, leading to calling errors in these sequences. HECTOR appears as a more optimized approach to deal with indels in homopolymers [16]. Although particularly powerful, this software is designed to correct genome-wide sequences, which implies improvements in runtimes but also a relative tolerance for false negatives, making it not adapted for gene-specific diagnosis purposes. In contrast, AmpliconNoise was designed to correct indel errors in PCR-based pyrosequences [18]. It is, however, computing-power demanding and it is hardly usable by nonbioinformaticians.

Our home-made software AGSA allows easily visualizing the data of each problematic homopolymer and quickly eliminating most of these false positive variants and generates easy-to-use results tables of validated amplicons and annotated variants.

Globally our proposed method of 454 sequencing is thus well adapted to constitutional sequencing diagnostics, since it is very sensitive, faster, and less expensive than Sanger sequencing.

## Supplementary Material

Figure S1: Screenshots of AGSA GUI (a) First interface with parameters to be adjusted before starting the analysis, including boundaries of the region of interest and read threshold values. Mouse hovers (or tooltips) give more details on the parameter to be set. Default parameters can be saved for each project. The duck with a yellow rugby suit and a broken leg (out of 3!) reminds that it has been developed in the home of ASM, a French rugby team, and that, although very useful for diagnosis purpose, there is nothing to write home about (« does not break three legs to a duck » in French) compare to other projects in the lab. (b) The second interface is used to validate variants in homopolymers. Amplicons coloured in green indicate that all nucleotides pass the required depth threshold. The different reported metrics help the user's decision-making, and the button (labelled (Hist… » on the screenshot) links to the histograms shown in Figure 2. Figure S2: Quality of nucleotide calling along 454 reads. Calculations were made on a randomly selected 10% of samples. On the left axis, a Q- score of Q20 is similar to di-deoxy sequencing quality, Q30 is the standard for Next Generation Sequencing. On the right axis, the green line represents the percentage of nucleotides with a Q-score above Q30 for each position along the read; the orange line represents the percentage of nucleotides with a Q-score above Q20 for each position along the read. Sudden drops of these lines correspond to the ends of amplicons: all nucleotides called after an amplicon is terminated have a quality of zero, lowering the average. After the end of the longest amplicon (501bp), the percentage of nucleotides with a good Q-score is null. Figure S3: Independent classification of AGSA histograms. 299 homopolymers in six locations were blindly assessed by two persons. 91% were concordant and most of the remaining cases were due to one reader being not sure how to assign the variant. Figure S4: 39 BRCA1 and 33 BRCA2 samples were analysed both with AGSA software and with SeqNext, using the same 20% threshold for variant calling. Both SeqNext and AGSA detected all 310 variants validated with the Sanger technique. AGSA reported 85 false positive variants and SeqNext 177.

## Figures and Tables

**Table 1:** Comparison of the variants detected by Sanger, by GS-Flx with SeqNext analysis, and by GS-Flx with AGSA analysis.

Gene	HGVS nomenclature	Sanger	AVA + AgsA	SeqNext
BRCA1	c.19-47del29	h	h (56%)^*∗∗*^	—^*∗∗*^
	c.81-12delC	—	—	h (41%)^†^
	c.124delA	h	h (48%)	h (48%)
	c.212+1G>A	h	h (72%)^*∗∗*^	—^*∗∗*^
	c.342-343delTC	h	h (36%)	h (37%)
	c.671-11dup	—	h (45%)^†^	h (43%)^†^
	c.798-799del	h	h (48%)	h (48%)
	c.1116G>A	h	h (43%)	h (43%)
	c.1390dupA	h	h (49%)	h (48%)
	c.1823-1826del	h	h (46%)	h (46%)
	c.1953_1956delGAAA	h	h (35%)	h (34%)
	c.2077G>A	h	h (60%)	h (62%)
	c.2082C>T	H	**H** (100%)	**H** (100%)
	c.2269delG	h	h (66%)	h (66%)
	c.2612C>T	h	h (42%)	h (42%)
	c.3113A>G	h	h (51%)	h (52%)
	c.3548A>G	h	h (49%)	h (49%)
	3839-3843del5ins4	h	h (52%)	h (52%)
	c.4127del	h	h (56%)	h (44%)
	c.4214-4215delIns5	—	—	h (23%)^†^
	c.4221delins9	—	—	h (26%)^†^
	C.4227-4237delins16	—	—	h (24%)^†^
	c.4243-4244delGA	—	—	h (26%)^†^
	c.4281_4282ins39	h	h (44%)	**—** ^††^
	c.4308T>C	h	h (55%)	h (49%)
	c.4575-4585del11	h	h (46%)	h (43%)
	c.4810C>T	h	h (58%)	h (53%)
	c.5266dupC	h	h (59%)	h (59%)
	c.5333-20_5333-19insT	—	—	h (25%)^†^

BRCA2	c.37_44del8	h	h (25%)^*∗∗*^	h (26%)
	c.1114A>C	h	h (47%)	h (51%)
	c.1246A>G	h	h (47%)	h (49%)
	c.1553_1554insT	—	h (31%)^*∗∗*†^	—^*∗∗*^
	c.1748_1749insA	—	h (47%)^*∗∗*†^	h (26%)^†^
	c.1759-1761delinsC	—	—	h (25%)^†^
	c.1774delT	—	—	h (33%)^†^
	c.1804-1806delins3	—	—	h (21%)^†^
	c.1803dupA	—	—	h (43%)^†^
	c.1815dupA	—	h (68%)^†^	h (31%)^†^
	c.1823dupA	—	—	h (33%)^†^
	c.1833dupA	—	—	h (21%)^†^
	c.2589T>A	—	—	h (34%)^†^
	c.2803G>A	h	h (39%)	h (40%)
	c.3479G>A	—	h (31%)^*∗∗*†^	—
	c.3807T>C	h	h (44%)	h (42%)
	c.4332-4333delTA	—	—	h (66%)^†^
	c.4350dupT	—	—	h (44%)^†^
	c.4781delins3	—	—	h (22%)^†^
	c.5073dupA	h	h (42%)	h (41%)
	c.5385dupA	—	—	h (22%)^†^
	c.5459_5460insA	—	h (32%)^*∗∗*†^	—
	c.7977-10dup	—	—	h (70%)^†^
	c.8125dupA	—	—	h (23%)^†^
	c.8147-8148insA	—	—	h (29%)^†^
	c.8574dup	—	h (38%)^†^	h (30%)^†^
	c.8797del	—	H^†^	—
	c.8800del	—	h (80%)^*∗∗*†^	—
	c.8823dupA	—	—	h (28%)^†^
	c.8946dup	—	h (27%)^*∗∗*†^	—
	c.10083del	—	—	h (21%)^†^
	c.10115dupC	—	—	h (50%)^†^
	c.10122delC	—	—	h (32%)^†^

False negative (out of 64)^††^		0 (reference)^††^	0^††^	1^†^
False positive (out of 64)^†^		0 (reference)^†^	10^†^	28^††^

% of reads carrying the variant is given in parentheses.

^*∗∗*^Depth of the variant was <40 reads.

^†^Cells highlight false positives; ^††^cells highlight false negatives.

## Data Access

Because supporting data are genomic data of patients, they must remain confidential and they cannot be made available to the scientific community. Other data supporting the results of this paper are included within the paper.
