# Supplementary figures and images for: Improved Efficiency and Reliability of NGS Amplicon Sequencing Data Analysis for Genetic Diagnostic Procedures Using AGSA Software
Source: Biomed Res Int. 2016 Aug 30;2016:5623089. doi: 10.1155/2016/5623089 (PMC5021467; doi:10.1155/2016/5623089)

Supplementary Figure 1

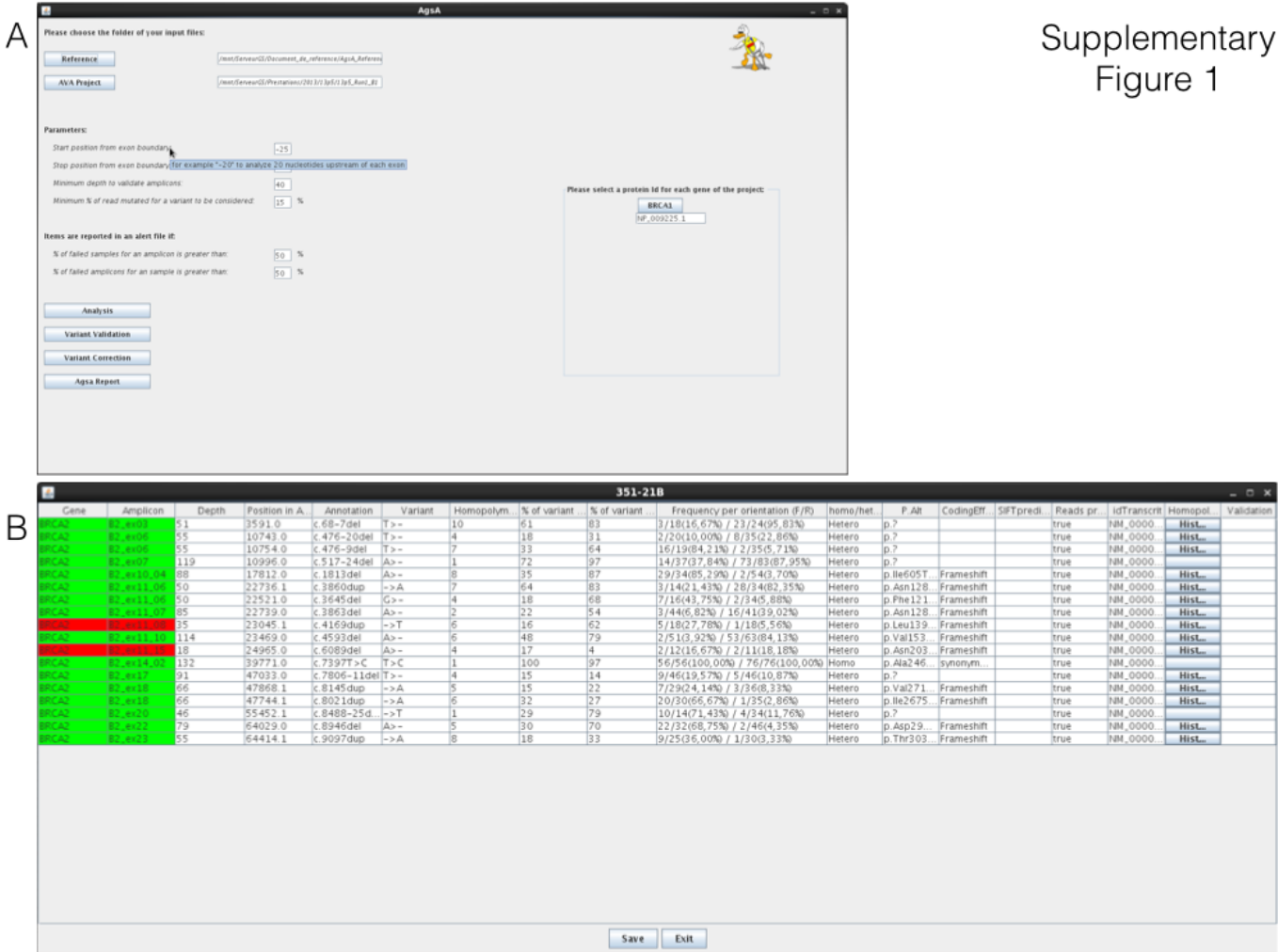

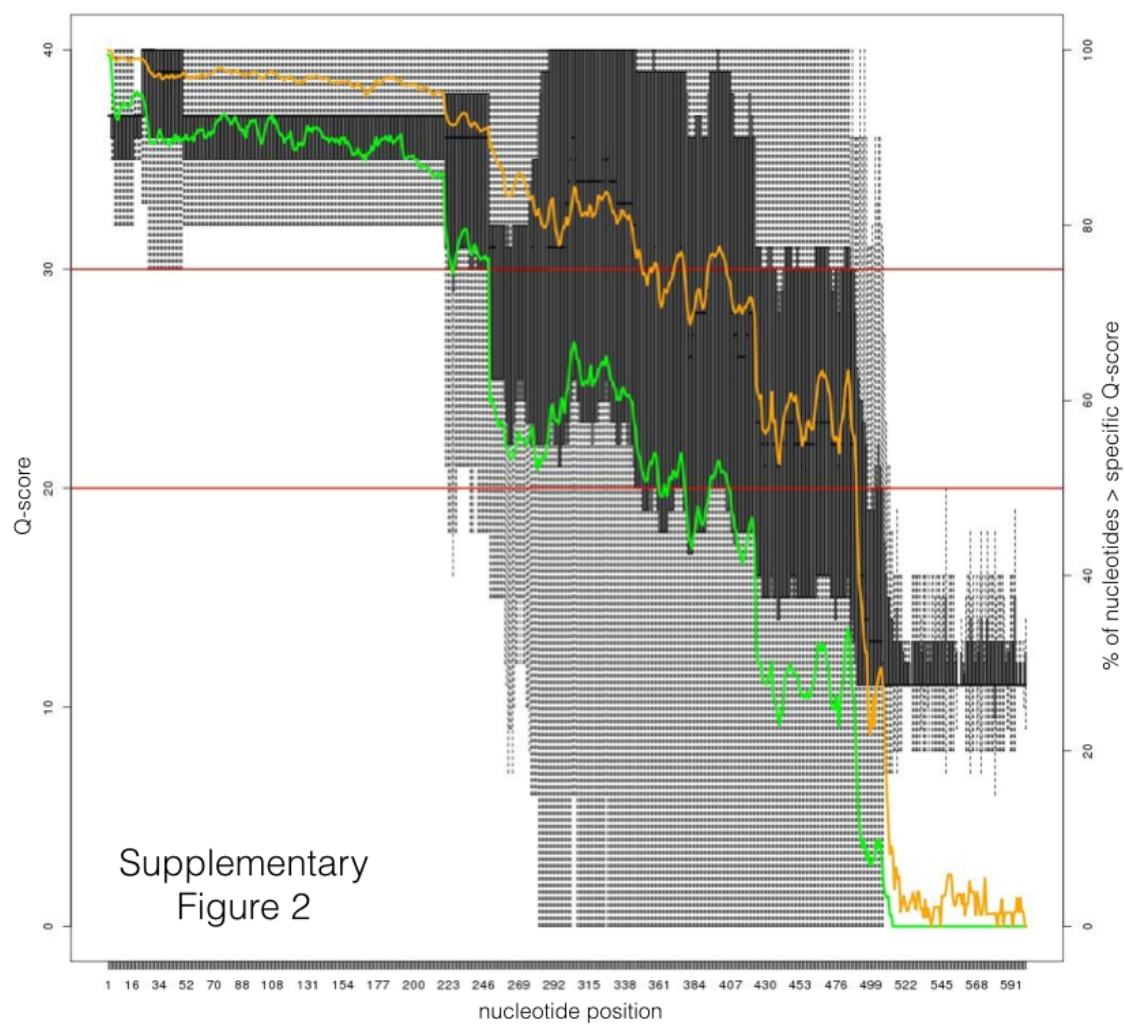

Supplementary  
Figure 3

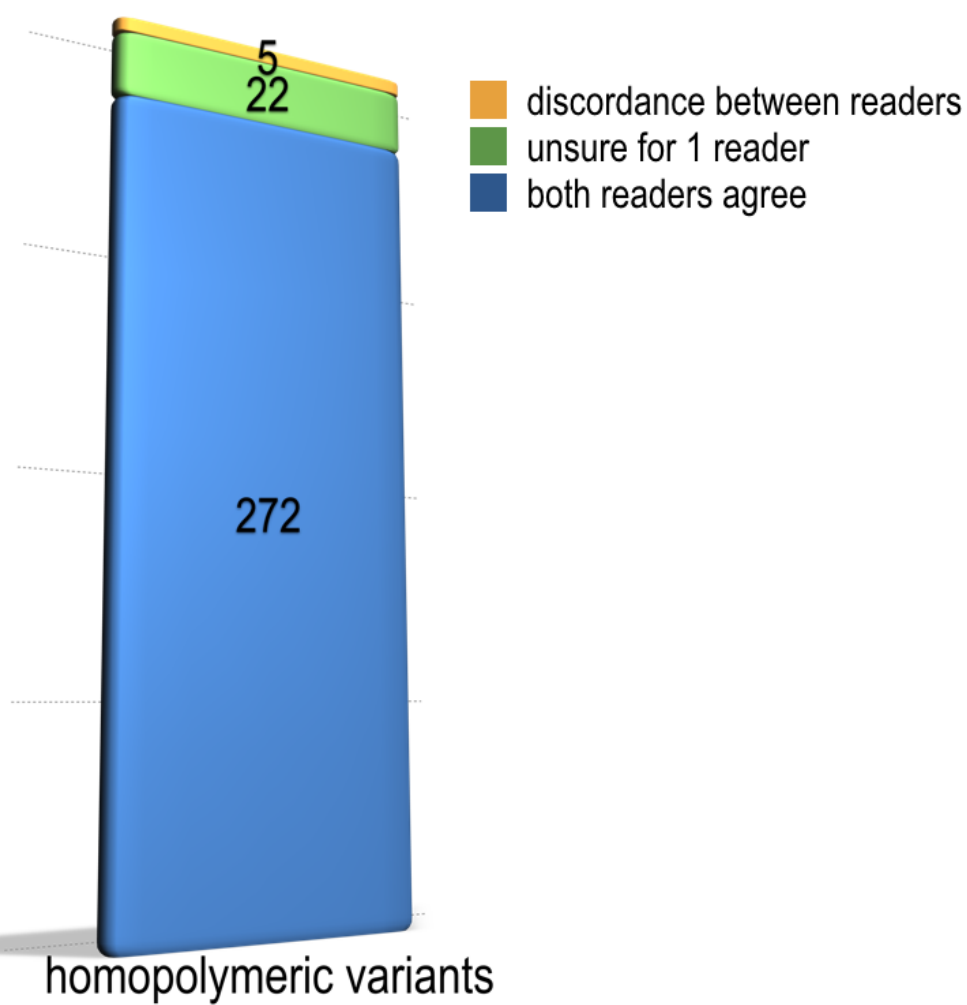

Supplementary  
Figure 4

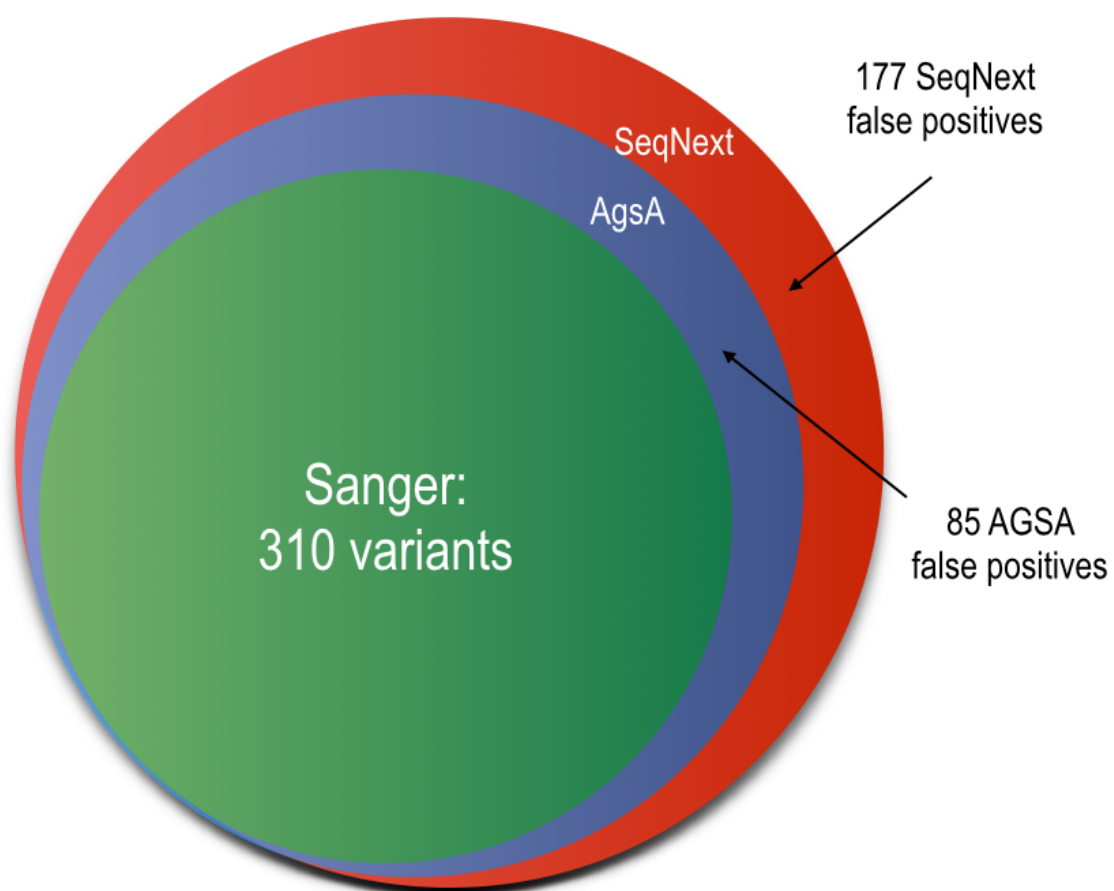

Supplement: Supplementary file 1 — Figure S1: Screenshots of AGSA GUI (a) First interface with parameters to be adjusted before starting the analysis, including boundaries of the region of interest and read threshold values. Mouse hovers (or tooltips) give more details on the parameter to be set. Default parameters can be saved for each project. The duck with a yellow rugby suit and a broken leg (out of 3!) reminds that it has been developed in the home of ASM, a French rugby team, and that, although very useful for diagnosis purpose, there is nothing to write home about (« does not break three legs to a duck » in French) compare to other projects in the lab. (b) The second interface is used to validate variants in homopolymers. Amplicons coloured in green indicate that all nucleotides pass the required depth threshold. The different reported metrics help the user's decision-making, and the button (labelled (Hist… » on the screenshot) links to the histograms shown in Figure 2. Figure S2: Quality of nucleotide calling along 454 reads. Calculations were made on a randomly selected 10% of samples. On the left axis, a Q- score of Q20 is similar to di-deoxy sequencing quality, Q30 is the standard for Next Generation Sequencing. On the right axis, the green line represents the percentage of nucleotides with a Q-score above Q30 for each position along the read; the orange line represents the percentage of nucleotides with a Q-score above Q20 for each position along the read. Sudden drops of these lines correspond to the ends of amplicons: all nucleotides called after an amplicon is terminated have a quality of zero, lowering the average. After the end of the longest amplicon (501bp), the percentage of nucleotides with a good Q-score is null. Figure S3: Independent classification of AGSA histograms. 299 homopolymers in six locations were blindly assessed by two persons. 91% were concordant and most of the remaining cases were due to one reader being not sure how to assign the variant. Figure S4: 39 BRCA1 and [file 5623089.f1.pdf]
